# Resonance Coupling in Si@WS_2_Core-Ω Shell Nanostructure

**DOI:** 10.3390/nano13030462

**Published:** 2023-01-23

**Authors:** Haomin Guo, Qi Hu, Chengyun Zhang, Zihao Fan, Haiwen Liu, Runmin Wu, Zhiyu Liu, Shusheng Pan

**Affiliations:** 1 School of Physics and Materials Science, Guangzhou University, Guangzhou 510006, China; 2 Research Center for Advanced Information Materials (CAIM), Huangpu Research and Graduate School of Guangzhou University, Guangzhou 510555, China

**Keywords:** laser–matter interaction, resonance coupling, Si@WS_2_ core-Ω shell nanostructure

## Abstract

Realizing strong laser–matter interaction in a heterostructure consisting of two-dimensional transition metal dichalcogenides (TMDCs) and an optical nanocavity is a potential strategy for novel photonic devices. In this paper, two core-Ω shell nanostructures, Si@WS_2_ core-Ω shell nanostructure on glass/Si substrates, are briefly introduced. A strong laser–matter interaction occurred in the Si@WS_2_ core-Ω shell nanostructure when it was excited by femtosecond (fs) laser in the near-infrared-1 region (NIR-1, 650 nm–950 nm), resulting in a resonance coupling between the electric dipole resonance (EDR) of the Si nanosphere (NS) and the exciton resonance of the WS_2_ nanomembrane (NMB). The generation of resonance coupling regulates the resonant mode of the nanostructure to realize the multi-dimensional nonlinear optical response, which can be utilized in the fields of biological imaging and nanoscale light source.

## 1. Introduction

The nanostructures of monocrystalline Si with different morphologies are of fundamental significance in optical diodes [1], resistive switching devices [2], microcavity filters [3] and other applications. The Si NS can be fabricated by using the fs laser [4,5,6,7,8,9,10]. In addition, the Si NS with a high refractive index can support Mie resonance [11,12,13,14,15], including magnetic dipole resonance (MDR), EDR, magnetic quadrupole resonance (MQR), electric quadrupole resonance (EQR) and other higher-order modes. The two/three-photon absorption (2/3PA) processes occur in the Si NS when the MDR of Si NS is excited by NIR-1 fs laser [16,17,18,19], resulting in an enhancement in the two/three-photon up-conversion luminescence (2/3PL). Due to the desirable applications of the multiphoton up-conversion luminescence (MPL) in photocatalysis [20], bioimaging [21,22,23] and nanoscale light source [24,25], researchers around the world focused on the MPL. For example, the red/NIR emissive carbon nanodots exhibit four-photon up-conversion luminescence (4PL) when they are excited by fs laser at the wavelength of 2000 nm [26]. However, the resonant modes of Si NS cannot be self-hybridized, resulting in the inability to provide multiple hybrid resonant modes. The core–shell nanostructures provide a strategy for resonant mode hybridization.

Moreover, the interaction between the laser and TMDCs can realize the light regulation, which is one of the research hotspots. For example, the WS_2_@Fe_3_O_4_ nanocomposites [27] can be used for imaging and therapy functionalities. At K and K’ points of hexagonal Brillouin zones, the monolayer WS_2_ with direct bandgap can realize the 2PA process [28,29]. The metal substrates could enhance the resonance coupling of the hybrid system [30]. Hence, the Si NS is often directly placed on the TMDCs NMB/Metal (Au, Ag, etc.) substrate to investigate the Fano resonance [31], the directional scattering enhancement [32] and so on. In the above nanostructures, the contact surface between the Si NS and the TMDCs NMB is small, resulting in a weak resonance coupling. In order to overcome this shortcoming, the construction of the Si@WS_2_ core-–shell nanostructure [33] is proposed to increase the contact area, which can enhance the resonance coupling [34,35,36] by matching the MDR of Si NS with the exciton resonance of the WS_2_ nanoshell. It is noticed that the previous studies mainly focused on resonance coupling between the MDR of Si NS and the exciton resonance of TMDCs NMB. However, few researchers have studied the resonance coupling between the exciton resonance of TMDCS NMB and other resonant modes of Si NS, particularly the EDR mode.

In this paper, a strong laser–matter interaction occurred in a Si@WS_2_ core-Ω shell nanostructure when it was excited by fs laser, resulting in a resonance coupling between the EDR of Si NS and the exciton resonance of WS_2_ NMB. The resonance coupling was induced by a wavelength matching the EDR of Si NS with the exciton resonant mode of WS_2_ NMB to regulate the optical response of the Si@WS_2_ core-Ω shell nanostructure. Experimentally, two systems were constructed to demonstrate the existence of resonance coupling in the Si@WS_2_ core-Ω shell nanostructure: the Si@WS_2_ core-Ω shell nanostructure on a glass substrate (system-1) and the Si@WS_2_ core-Ω shell nanostructure on a Si substrate (system-2).

## 2. Materials and Methods

### 2.1. Sample Preparation

Si NSs with different diameters were fabricated using the fs laser-induced-backward-transfer (LIBT) technology [37,38]. A Si wafer (thickness: 525 *µm*, orientation: 111, type: N, Dopant: Sb, Ningbo Sibranch Microelectronics Technology CO., LTD., Ningbo, China) covered with a glass (MATSUNAMI, thickness: 130 *µm*) was placed on the 3D electronic translation platform controlled by a computer. The fs laser delivered by a fs amplifier (Legend Elite, Coherent, San Francisco, CA, USA, central wavelength: 800 nm, pulse duration: 100 fs, repetition rate: 1kHz, power: 4 mW) was focused on the surface of the Si substrate. The induced Si NSs were partly sprayed onto the glass substrate, and the rest of the Si NSs remained on the surface of the Si substrate. The WS_2_ NMB (WS_2_ Target Material, Purity: 99.9%, Diameter: 50.8 mm, Zhongnuo Advanced Material(Beijing)Technology CO., LTD., Beijing, China) was sputtered onto the surface of the Si NS on glass/Si substrates to form the system-1/-2 using the magnetron sputter coating technique (JCP-350, Beijing Technol Science CO., LTD., Beijing, China).

### 2.2. Morphology Characterization and Spectroscopy Measurement

The scattering and luminescence spectra contain three different stages (stage 1: Si NS on glass/Si substrates; stage 2: system-1/-2; stage 3: system-1/-2 (annealed)), which were measured by using a dark-filed optical microscope (BX53, Olympus, Tokyo, Japan) equipped with a spectrometer (SR-500i-B1-R, Andor) and a color charge coupled device (CCD) (01-QIClick-R-F-CLR-12, QIMAGING, Surrey, BC, Canada). The 100× objective lens with a numerical aperture of 0.8 was utilized to collect the scattering light and the luminescence signal excited by an fs oscillator (Mira-HP, Coherent, San Francisco, CA, USA). The integration time for the scattering spectra measurement was 0.6 s. The integration time for the luminescence spectra measurement was 2 s and the gain coefficient was set to 20 times. The surface morphology and the element mapping distribution images of the samples were analyzed using the scanning electron microscope (SEM, ZEISS Ultra 55, Carl Zeiss, Oberkochen, Germany) and energy-dispersive spectroscopy (EDS, ZEISS Ultra 55, Carl Zeiss, Oberkochen, Germany). The crystallinity of the sample was analyzed using the transmission electron microscope (TEM, JEM-2100F, JEOL, Tokyo, Japan).

### 2.3. The Scattering Cross-Section

Firstly, two 3D models (Si NS on the glass/Si substrates) were established using the Finite Element Analysis (FEA) method. Secondly, the Ω-type WS_2_ NMB was placed on the above two models. The diameter of the Si NS in the two models was 170 nm and the thickness of the WS_2_ NMB was 9 nm, so the total diameter of the core-–shell nanostructure was 188 nm. The glass/Si substrates were 100 nm. The mesh size of the two models, which was as small as 1 nm, was used to ensure the convergence of the numerical simulations. The refractive index of the WS_2_ ($n_{WS_{2}}$) [39] and Si ($n_{Si}$) [40] was referred to references [39,40]. The refractive index of the Si and WS_2_ is shown in Figure S1. When the Si@WS_2_ core-Ω shell nanostructure was established, we considered the fact that we were dealing with lossy and dispersive nanocavities. The refractive index of the Si NS and the WS_2_ NMB contained the real and the imaginary parts. The real part represented the dispersion of the electromagnetic wave by the medium, and the imaginary part represented the absorption of the electromagnetic wave by the medium. The Si@WS_2_ core-Ω shell nanostructure was a nanocavity with loss and dispersion. The surrounding refractive index ($n_{m}$) was chosen to be 1. The calculated results are basically matched with the experimental structure.

Moreover, the EDR, MDR, EQR and MQR modes of the Si@WS_2_ core-Ω shell nanostructure in the free space were calculated according to the Lorenz–Mie theory [41,42,43]: $\sigma_{sca}=\frac{2\pi }{k^{2}}\overset{\infty }{\underset{n=1}{\sum }}\left(2n+1\right)\left({\left|a_{n}\right|}^{2}+{\left|b_{n}\right|}^{2}\right)$, where the $k=\left(2\pi n_{m}\right)/\lambda$ and $n_{m}$ is the refractive index of the environment. Based on the Lorenz–Mie theory, $a_{n}$ and are the electric and magnetic mode coefficients of the core-–shell nanostructure in the free space, respectively. For the core-–shell nanostructure (core radius a, (x=k×a) and outer radius b, (y=k×b)) with core refractive index ($m_{1}$) and nanoshell refractive index ($m_{2}$), Lorenz–Mie coefficients ($a_{n}$ and $b_{n}$) are calculated by using the modified relations:

$$a_{n}=\frac{\psi_{n}\left(y\right)\left[\psi_{n}^{\prime }\left(m_{2}y\right)-A_{n}\chi_{n}^{\prime }\left(m_{2}y\right)\right]-m_{2}\psi_{n}^{\prime }\left(y\right)\left[\psi_{n}\left(m_{2}y\right)-A_{n}\chi_{n}\left(m_{2}y\right)\right]}{\xi_{n}\left(y\right)\left[\psi_{n}^{\prime }\left(m_{2}y\right)-A_{n}\chi_{n}^{\prime }\left(m_{2}y\right)\right]-m_{2}\xi_{n}^{\prime }\left(y\right)\left[\psi_{n}\left(m_{2}y\right)-A_{n}\chi_{n}\left(m_{2}y\right)\right]}$$

$$b_{n}=\frac{m_{2}\psi_{n}\left(y\right)\left[\psi_{n}^{\prime }\left(m_{2}y\right)-B_{n}\chi_{n}^{\prime }\left(m_{2}y\right)\right]-\psi_{n}^{\prime }\left(y\right)\left[\psi_{n}\left(m_{2}y\right)-B_{n}\chi_{n}\left(m_{2}y\right)\right]}{m_{2}\xi_{n}\left(y\right)\left[\psi_{n}^{\prime }\left(m_{2}y\right)-B_{n}\chi_{n}^{\prime }\left(m_{2}y\right)\right]-\xi_{n}^{\prime }\left(y\right)\left[\psi_{n}\left(m_{2}y\right)-B_{n}\chi_{n}\left(m_{2}y\right)\right]}$$

The functions ($\psi_{n}$, $\chi_{n}$ and $\xi_{n}$) are related to Riccati–Bessel functions ($\psi_{n}\left(\rho \right)=\rho j_{n}\left(\rho \right)$, $\chi_{n}\left(\rho \right)=\rho n_{n}\left(\rho \right)$, $\xi_{n}\left(\rho \right)=\rho h_{n}^{1}\left(\rho \right)$), where $j_{n}\left(\rho \right)$ is the spherical Bessel function, $n_{n}\left(\rho \right)$ is the spherical Neumann function and $h_{n}^{1}\left(\rho \right)$ is the Hankel function of the first kind. Since the refractive indexes of the core and nanoshell are different, $A_{n}$ and $B_{n}$ correlate the refractive indexes of the core and nanoshell, showing the existence of the interaction between the core and nanoshell.

$$A_{n}=\frac{m_{2}\psi_{n}\left(m_{2}x\right)\psi_{n}^{\prime }\left(m_{1}x\right)-m_{1}\psi_{n}^{\prime }\left(m_{2}x\right)\psi_{n}\left(m_{1}x\right)}{m_{2}\chi_{n}\left(m_{2}x\right)\psi_{n}^{\prime }\left(m_{1}x\right)-m_{1}\chi_{n}^{\prime }\left(m_{2}x\right)\psi_{n}\left(m_{1}x\right)}$$

$$B_{n}=\frac{m_{2}\psi_{n}\left(m_{1}x\right)\psi_{n}^{\prime }\left(m_{2}x\right)-m_{1}\psi_{n}\left(m_{2}x\right)\psi_{n}^{\prime }\left(m_{1}x\right)}{m_{2}\chi_{n}^{\prime }\left(m_{2}x\right)\psi_{n}\left(m_{1}x\right)-m_{1}\psi_{n}^{\prime }\left(m_{1}x\right)\chi_{n}\left(m_{2}x\right)}$$

### 2.4. The Enhancement Factor

The enhancement factor was calculated with the formula: $I=\frac{1}{V}\int_{nanostructure}^{}{\left|E\left(\lambda ,r\right)/E_{0}\right|}^{2}dV$.$E_{0}$ is the electric field amplitude of the incident laser. E(λ,r) is the electric field amplitude of the system-1 calculated using FEA method. ${\left|E\left(\lambda ,r\right)/E_{0}\right|}^{2}$ is calculated by using the volume integral to get the enhancement factor.

## 3. Results

### 3.1. Morphology Characterization of Si@WS_2_Core-Ω Shell Nanostructure

**Figure 1:**
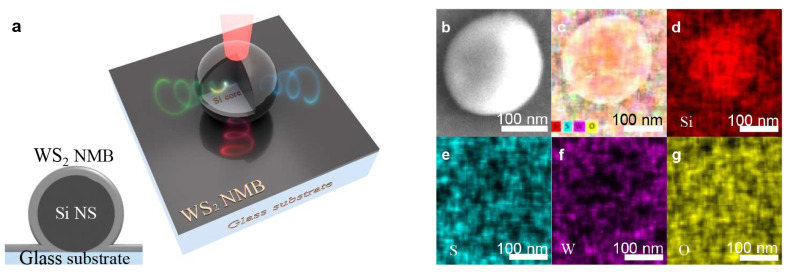
(**a**) Schematic diagram of the system-1; the inset shows the cross-section of the Ω-shape shell nanostructure. (**b**) The SEM image of the system-1 (annealed) with d ~190 nm. (**c**) The EDS mapping images of superposition of Si (**d**), S (**e**), W (**f**), O (**g**) elements.

Firstly, the Si NSs on glass substrate were fabricated by using the fs LIBT technology, and then the WS_2_ NMB was sputtered on the surface of the Si NS on glass substrate using the magnetron sputtering technique (Table S1) to construct the system-1. The schematic diagram of the system-1 is shown in Figure 1a.

The inset in Figure 1a shows the cross-section of the core-Ω shell nanostructure. The SEM image of the system-1 (annealed, Figure 1b) shows that the sample is a sphere with a diameter of (d) ~190 nm. Figure 1c shows the EDS mapping image of the superposition of each element in the system-1 (annealed). In detail, the Si element (Figure 1d) is obvious in the core area, while the sulfur (S, Figure 1e), tungsten (W, Figure 1f) and oxygen (O, Figure 1g) elements are distributed in the whole plane. In addition, the SEM image of 45° view is shown in Figure S2.

### 3.2. Optical Response of Si@WS_2_Core-Ω Shell Nanostructure

**Figure 2:**
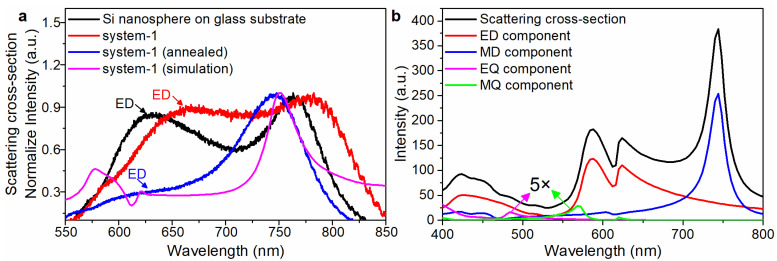
(**a**) The forward scattering cross-section curves of the Si NS on glass substrate, the system-1, the system-1 (annealed) and the simulated result are shown in the black, red, blue and pink lines, respectively; the simulated forward scattering cross-section curve (pink line) is calculated using FEA method. (**b**) The scattering cross-section curves of the Si@WS_2_ core-Ω shell nanostructure in the free space were calculated using the Lorenz–Mie theory.

Before laser annealing, the forward scattering cross-section curve (Figure 2a, red line) of the system-1 was similar to that (Figure 2a, black line) of the Si NS on glass substrate, and both had obvious EDR characteristic peaks. According to the TEM image, it is observed that the initial WS_2_ NMB is amorphous (Figure S3a). This is because the crystallinity of the material [44] has great influence on the nonlinear response, resulting in only weak resonance coupling between the Si NS and the WS_2_ NMB. After annealing, the crystallinity of the WS_2_ NMB increased gradually (Figure S3b). 

An fs laser with suitable power was utilized to continuously irradiate system-1 to achieve the annealing effect. Obviously, after laser annealing, the intensity of the EDR characteristic peak of the system-1 (annealed) was decreased dramatically (Figure 2a, blue line). The simulated forward scattering cross-section curve of the system-1 calculated using FEA method (Figure 2a, pink line) also shows that the intensity of the EDR characteristic peak was dramatically decreased (see another sample in Figure S4).

In contrast to plasmonic nanoparticles, the first-order resonance mode of Si@WS_2_ core-Ω shell nanostructure is magnetic dipole (MD) resonance, which occurs when the wavelength of light inside the dielectric nanoparticles equals the diameter [14]: $\lambda_{MD}=2\times R_{Si@WS_{2}}\times n_{Si@WS_{2}}$.

**Figure 3:**
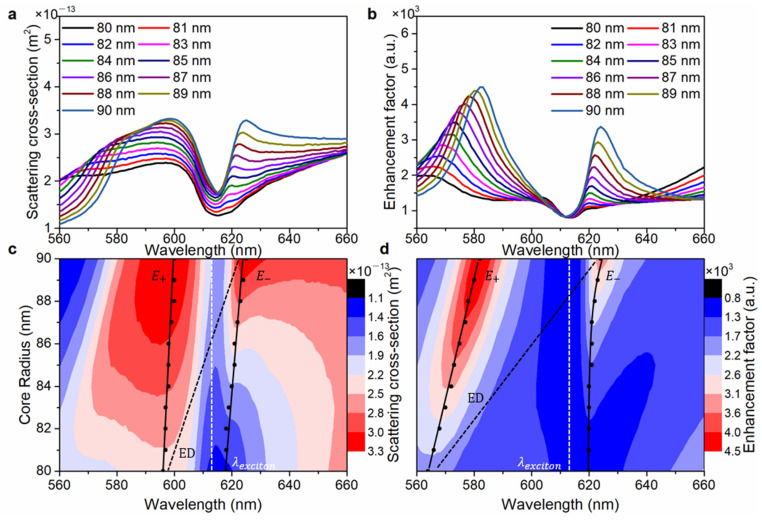
(**a**) Simulated scattering cross-section curves of system-1 with the different silicon radii. (**b**) Simulated enhancement factor intensity curves of system-1 with the different silicon radii. (**c**) Simulated scattering cross-section map of system-1 varying with wavelength and core radius. (**d**) Simulated enhancement factor intensity map of system-1 varying with wavelength and core radius.

The EDR (Figure 2b, red line), MDR (Figure 2b, blue line), EQR (Figure 2b, pink line) and MQR (Figure 2b, green line) components of the Si@WS_2_ core-Ω shell nanostructure were calculated by using the Lorenz–Mie theory (the detailed derivation in the numerical simulation section). The EDR mode component of the scattering cross-section appears as a resonant valley at the wavelength of 612 nm. As shown in Figure 3, by changing the core radius of system-1, the EDR wavelength sweeps over the wavelength of the exciton transition to alter the degree of interaction of the two resonators. The resonant valley appears at the wavelength of 612 nm (Figure 3a). The 2D map of the scattering cross-section of system-1(Figure 3c) shows that anti-crossing behavior can be seen when the EDR wavelength spectrally intersects the exciton resonance (white dashed line). Meanwhile, it also shows that the EDR scattering peak splits into higher ($E_{+}$) and lower ($E_{-}$) energy branches at the position of the exciton resonance. The enhancement factor spectra of system-1 also show the existence of the resonant valley (612 nm, Figure 3b,d).

**Figure 4:**
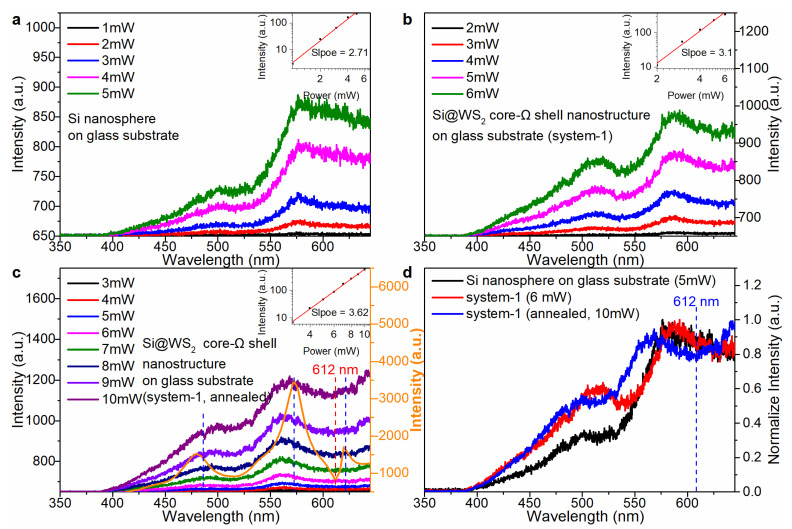
The dependence of luminescence on the excitation pulse power at three different stages. (**a**) The Si NS on glass substrate, excitation wavelength-777 nm. (**b**) The system-1, excitation wavelength-783 nm. (**c**) The system-1 (annealed) with d ~ 190 nm, excitation wavelength-773 nm; the simulated enhancement factor curve of system-1 (orange line) was calculated using FEA method. (**d**) Normalized luminescence comparison diagram at three different stages.

Thereafter, the luminescence spectra of system-1 were also measured to prove that resonance coupling occurs in the system-1. The dependence of the up-converted luminescence intensity on the excitation pulse power was plotted in a double-logarithmic coordinate to extract the slopes at three different stages. Before annealing, the slopes of the Si NS on glass substrate and the system-1 were 2.71 (Figure 4a, the inset) and 3.1 (Figure 4b, the inset), respectively. The slope of the Si NS on glass substrate was 2.71, close to 3, indicating that the up-converted luminescence was dominated by 3PL. Before annealing, the WS_2_ NMB weakly resonated with Si NS because WS_2_ NMB was amorphous, so the slope of the system-1 was 3.1. After annealing, the strong resonance coupling occurred in system-1 because the crystallinity of WS_2_ NMB was improved. The slope of the system-1 (annealed) increased to 3.62 (Figure 4c), indicating that the up-converted luminescence of system-1 (annealed) was 3/4PL. In addition, the enhancement factor curve (Figure 4c, orange line) showed strong gains at the wavelengths of 480 nm, 575 nm and 622 nm, basically matching the luminescence characteristic peaks of the system-1 (annealed). According to the comparison diagram of the normalized luminescence spectrum (Figure 4d), after annealing of system-1, the resonant valley was obvious at the wavelength of 612 nm (see another sample in Figure S5).

**Figure 5:**
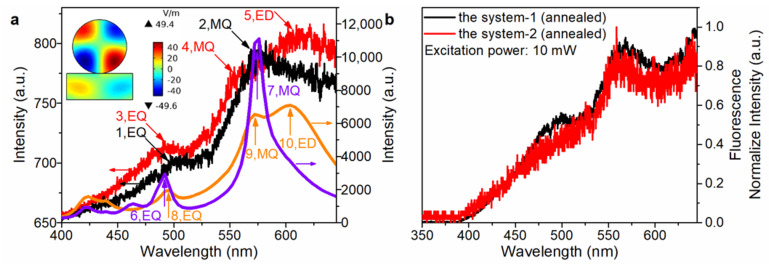
(**a**) The luminescence curves of Si NS on glass/Si substrates at an excitation wavelength of 777/775 nm and an excitation power of 5mW/14mW are shown as the black/red line; the simulated enhancement intensity curves of Si NS on glass/Si substrates are shown as the purple/orange; and the inset is the z component of electric filed in YZ plane at peak 10. (**b**) The comparison of normalized luminescence curves of the system-1/2 (annealed) at the same excitation power.

It is known that Si NS on different substrates has different optical responses, such as the optical anapole mode [45], etc. In this paper, it was found that the EDR of the Si NS was enhanced when the glass substrate was replaced by the Si substrate. The luminescence curve of the Si NS on glass substrate (Figure 5a, black line) showed two distinctly characteristic peaks, including the EQR (peak 1) and MQR (peak 2) characteristic peaks. The luminescence curve of the Si NS on Si substrate (Figure 5a, red line) also had EQR (peak 3) and MQR (peak 4) characteristic peaks. However, it is noticed that the luminescence curve of the Si NS on Si substrate had a special characteristic peak (peak 5). By comparing the enhancement factor curves of Si NS on glass (Figure 5a, purple line) and Si (Figure 5a, orange line) substrates, a characteristic peak (peak 10) was also found in the enhancement factor curve of the system-2. In addition, the inset (Figure 5) indicates that peak 10 is the EDR characteristic peak. Obviously, the luminescence curves of system-1/-2 (annealed) also have the resonant valley at the wavelength of 612 nm (Figure 5b). Hence, the resonance coupling occurs in the two systems. The detailed analysis of the system-2 is in Appendix B of Supplementary Materials (Figures S6–S9).

### 3.3. Electric Field Distribution of Si@WS_2_ Core-Ω Shell Nanostructure

**Figure 6:**
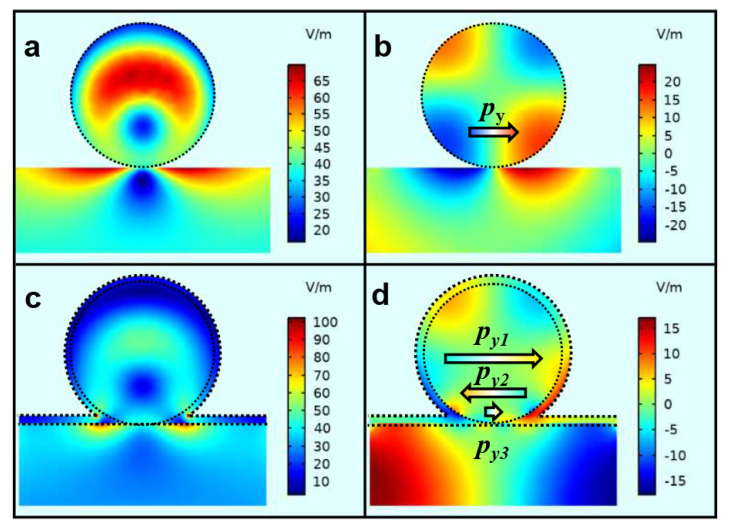
The simulated electric field distribution images. Distributions of electric field (**a**) and z component of electric field (**b**) of the Si NS on glass substrate in YZ plane at the wavelength of 612 nm. Distributions of electric field (**c**) and z component of electric field (**d**) of the system-1 in YZ plane at the same wavelength.

The luminescence spectra of two systems (annealed) display the resonant valley at the wavelength of 612 nm. The electric field distribution images of system-1 were plotted to analyze resonant modes of Si@WS_2_ core-Ω shell nanostructure. The electric field distribution images of Si NS on glass substrate and system-1 are plotted in Figure 6. By comparing the electric field of the Si NS on glass substrate (Figure 6a) with that of system-1 (Figure 6c), it was found that the electric field distribution of system-1 obviously changed, because the WS_2_ NMB was added to the Si NS on glass substrate to form the Si@WS_2_ core-Ω shell nanostructure. Further, the resonant mode of the Si NS on glass substrate was the EDR ($p_{y}$) mode at the wavelength of 612 nm (Figure 6b). The $p_{y}$ mode of Si NS was divided into three new modes ($p_{y1}$, $p_{y2}$ and $p_{y3}$) in the system-1 (Figure 6d). Hence, the resonance coupling between the EDR of Si NS and the exciton resonance of WS_2_ NMB occurred in the Si@WS_2_ core-Ω shell nanostructure.

## 4. Conclusions

In summary, there is a strong laser–matter interaction in the Si@WS_2_ core-Ω shell nanostructure excited by NIR-1 fs laser, resulting in resonance coupling between Si NS and WS_2_ NMB. The coupling effect was induced by the wavelength matching the EDR of Si NS with the exciton resonance of WS_2_ NMB, making the EDR mode of the Si NS split into three new modes in the Si@WS_2_ core-Ω shell nanostructure. The resonance coupling between the EDR of Si NS and the exciton resonance of the WS_2_ NMB dramatically reduced the EDR scattering cross-section peak to realize the regulation of resonant mode. The 3/4PL emission of the Si@WS_2_ core-Ω shell nanostructure was realized by the resonance coupling. Our study provides a new idea for the study of integrated and compatible nanostructured light sources based on the semiconductor NS@TMDCs NMB core-Ω shell nanostructures, which can achieve multi-dimensional nonlinear optical responses.

## Data Availability

Data underlying the results presented in this paper are not publicly available at this time, but may be obtained from the authors upon reasonable request.

## Supplementary Materials

The following supporting information can be downloaded at: https://www.mdpi.com/article/10.3390/nano13030462/s1. Figure S1 contains the refractive index images of Si and WS_2_. Figure S2 contains the SEM image of the system-1 (annealed, the first sample) at the 45° angle. Figure S3 contains the TEM images of initial/annealed WS_2_ NMB. Figure S4 contains the forward scattering cross-section spectra at three different stages (the second sample of system-1). Figure S5 contains the dependence of the nonlinear response spectra on excitation pulse power at three different stages (the second sample of system-1). Figure S6 contains the SEM and EDS images of the system-2 (annealed, the first sample). Figure S7 contains the dependence of the nonlinear response spectra on excitation pulse power at three different stages (the first sample of system-2). Figure S8 contains the simulated electric field distribution images of system-2. Figure S9 contains the dependence of the nonlinear response spectra on excitation pulse power at three different stages (the second sample of system-2). Table S1 contains the parameters for magnetron sputtering.
