# Supplementary material for: Resonance Coupling in Si@WS2Core-Ω Shell Nanostructure
Source: Nanomaterials (Basel). 2023 Jan 23;13(3):462. doi: 10.3390/nano13030462 (PMC9920409; doi:10.3390/nano13030462)
Supplement: Supplementary file 1 [file nanomaterials-13-00462-s001.zip › nanomaterials-2180094-supplementary.pdf]

Supporting information

Resonance coupling in Si@WS_2 core- Ω shell nanostructure

Haomin Guo [1], Qi Hu [1,2], Chengyun Zhang [1,2,*], Zihao Fan [1], Haiwen Liu [1], Runmin Wu [1], Zhiyu Liu [1,2] and Shusheng Pan [1,2,*]

[1] School of Physics and Materials Science, Guangzhou University, Guangzhou 510006, China.
[2] Research Center for Advanced Information Materials (CAIM), Huangpu Research and Graduate School of Guangzhou University, Guangzhou 510555, China.
*Corresponding: chyzhang@gzhu.edu.cn (C.Z.), sspan@gzhu.edu.cn (S.P.)

Appendix A

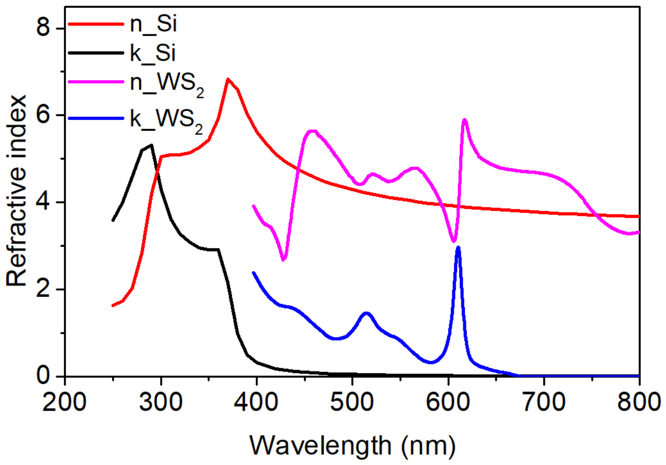

Figure S1. The real part refractive index of Si (red line) and WS_2 (pink line); the imaginary part refractive index of Si (black line) and WS_2 (blue line). The data are extracted from Ref [39,40].

Table S1 lists the parameters for fabrication of the core-Ω shell nanostructures by sputtering the WS_2 NMB onto the Si NS on glass/Si substrate under argon atmosphere.

Table S1. The parameters for magnetron sputtering.

Temperature	Power	Vacuum	Argon working pressure
60 °C	30 W	4×10^{-3} Pa	0.5 Pa

Top view SEM image of the system-1 (annealed) is shown in the Figure 2a. In order to better demonstrate Ω-type nanostructure, Figure S2 shows the SEM image at 45° angle.

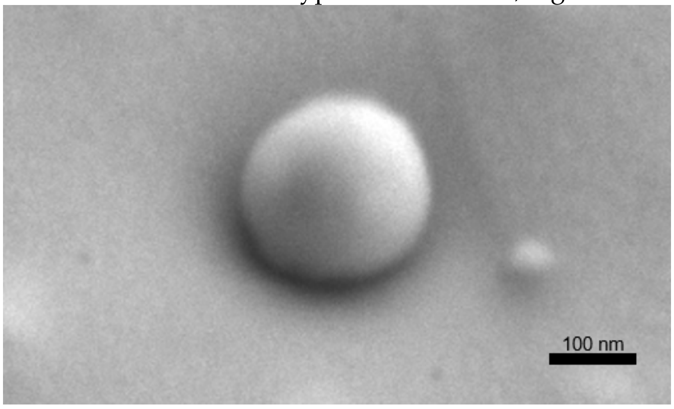

Figure S2. The SEM image of the system-1 (annealed) at the 45° angle.

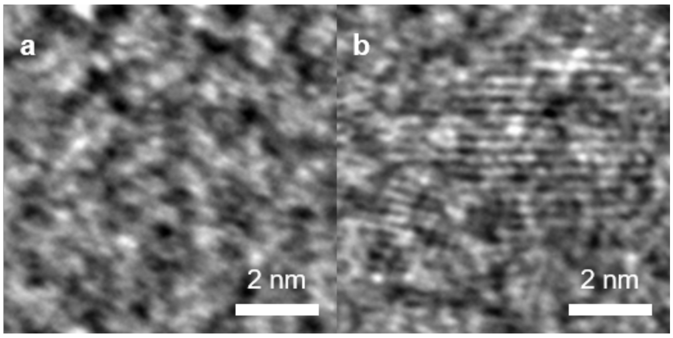

Figure S3. (a) TEM image of the initial WS_2 NMB. (b) TEM image of the WS_2 NMB (annealed).

A coupling property of the second sample corresponding to the system-1 is investigated. Before annealing, there is only weak resonance coupling between the WS_2 NMB and the Si NS. The EDR scattering characteristic peaks of Si NS on glass substrate (Figure S4, black line) and the system-1 (Figure S4, red line) are obvious. However, the EDR scattering characteristic peak decreases dramatically (Figure S4, blue line) when the system-1 is annealed. The dependence of the up-converted luminescence on the excitation pulse power is plotted in a double-logarithmic coordinate to get the slopes at three different stages. Before annealing, the extracted slope of the Si NS is 2.96 (Figure S5a), close to 3, indicating that the emission of 3PL is dominated. And the slope of the system-1 is 3.47 (Figure S5b) because there is a weak resonance coupling between the Si NS and coating WS_2 NMB.

After annealing, the crystallinity of the WS_2 NMB is improved, resulting in a strong resonance coupling between the Si NS and the WS_2 NMB. The slope of the system-1 (annealed) increases to 3.70 (Figure S5c), indicating that the luminescence emission of the system-1 (annealed) is dominated by 3/4PL. And the inset (Figure S5c) shows the surface morphology of the second sample of the system-1 (annealed) with d ~ 200 nm. The luminescence curve of the system-1 (annealed, Figure S5d, blue line) also shows the resonant valley at the wavelength of 612 nm.

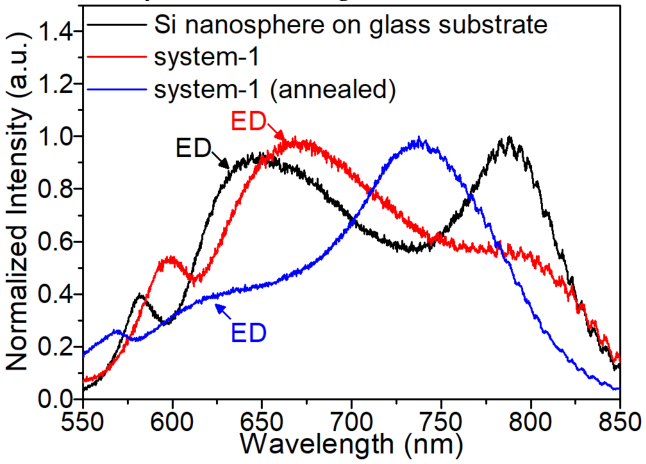

Figure S4. The forward scattering cross-section curves of the Si NS on glass substrate, the system-1, the system-1 (annealed) are shown as black, red, blue line, respectively.

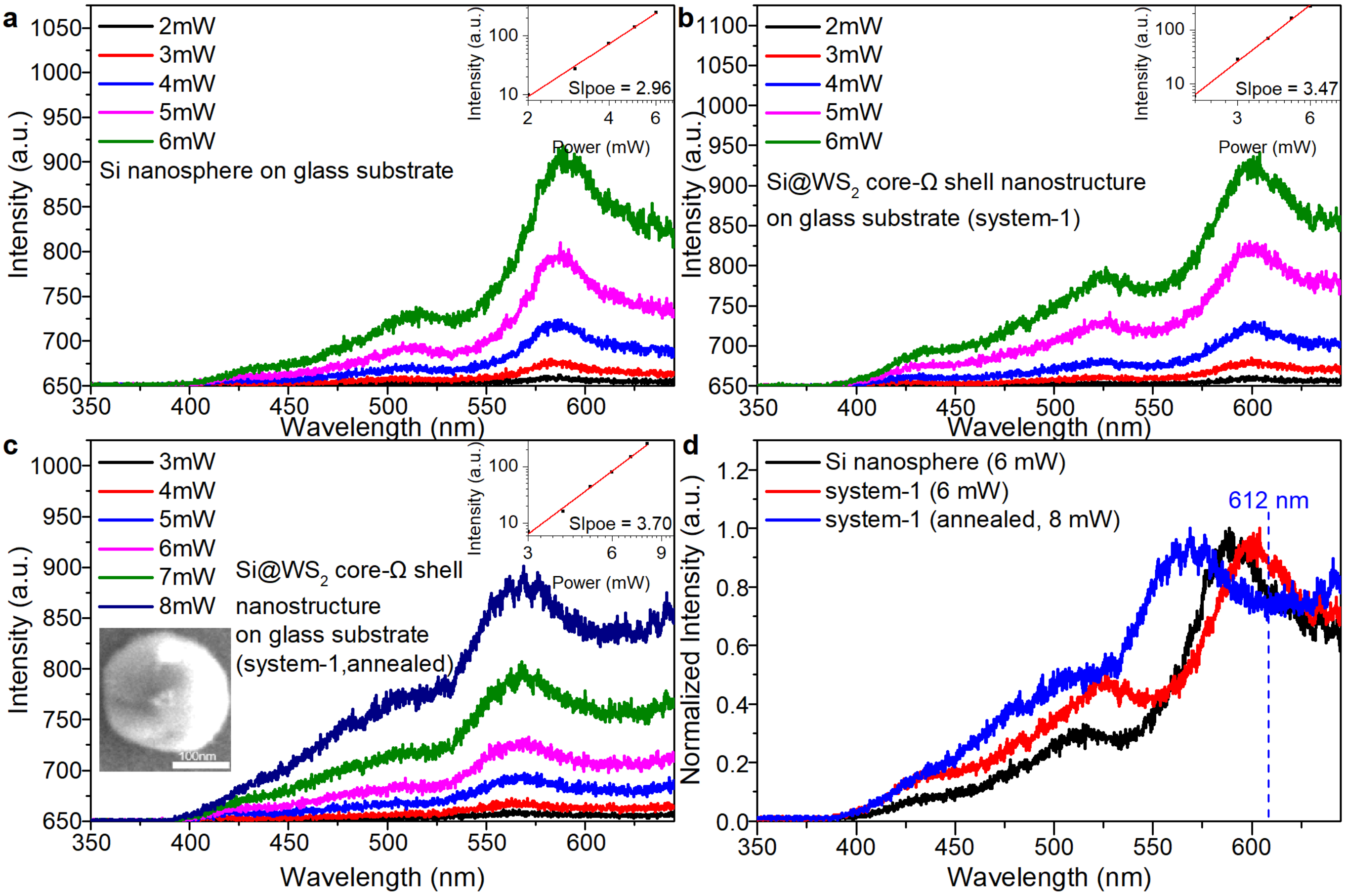

Figure S5. The dependence of the luminescence on excitation pulse power at three different stages. (a) the Si NS on glass substrate, excitation wavelength-787 nm. (b) the system-1, excitation wavelength-787 nm. (c) the system-1 (annealed) with d ~ 200 nm, excitation wavelength-773 nm. (d) Normalized luminescence comparison at three different stages.

Appendix B: the detailed analysis of the system-2

Figure S6a shows the SEM image of the system-2 (annealed) with d ~ 210 nm. The EDS image of elements superposition is shown in Figure S6b. Meanwhile, The Si element (Figure S6c) is obvious in the core area of the core-Ω shell nanostructure, and the sulfur (S, Figure S6d), tungsten (W, Figure S6e) and oxygen (O, Figure S6f) elements are uniformly distributed throughout the plane.

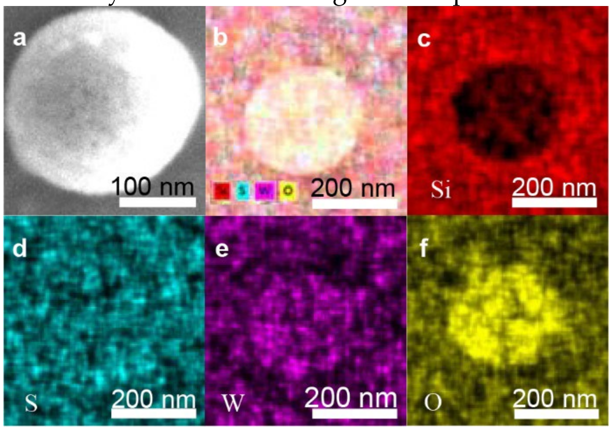

Figure S6. (a) the SEM image of the system-2 (annealed). (b) the EDS superposition diagram of Si (c), S (d), W (e), O (f) elements.

The dependence of the up-converted luminescence on the excitation pulse power is plotted in a double-logarithmic coordinate to get the slopes at three different states (Figure S7). Before annealing, the extracted slope of Si NS on Si substrate is 2.52 (Figure S7a). And

then, there is weak resonance coupling between the WS_2 NMB and the Si NS when the system-2 is excited by fs laser, resulting in the slope of 2.77 in the system-2 (Figure S7b). However, after annealing, an obvious resonant valley appears at the wavelength of 612 nm (Figure S7c), and the slope of the system-2 increases to 3.11 due to strong resonance coupling between the Si NS and WS_2 NMB. The luminescence spectra at three different stages are compared by normalization (Figure S7d).

The electric field distribution image of the Si NS on Si substrate is shown at Figure S8a. However, the electric field distribution of system-2 is obviously changed (Figure S8c). Because the WS_2 NMB is added to the Si NS on Si substrate to form the system-2. The z component of electric filed distribution contains the evidence of the strong laser-matter interaction. At the wavelength of 612 nm, the resonance mode of the Si NS on glass substrate is the EDR mode. However, the resonance coupling between the Si NS and the Si substrate results in dramatic change of EDR mode (Figure S8b). Hence, the resonance coupling between the Si NS and the WS_2 NMB in the system-2 is not obvious (Figure S8d) because of the influence of Si substrate.

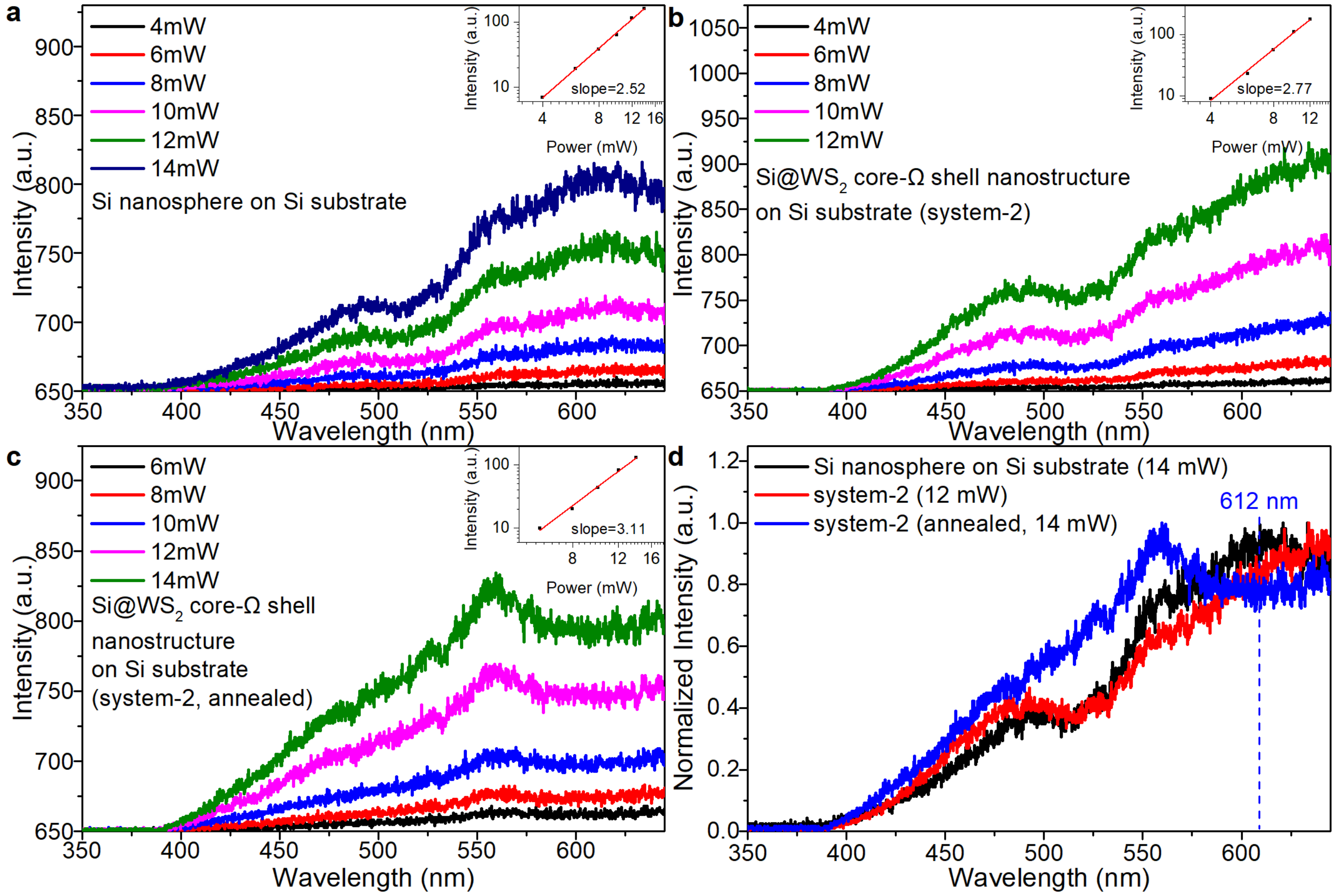

Figure S7: The dependence of the luminescence on excitation pulse power at three different stages. (a) the Si NS on Si substrate, excitation wavelength-775 nm. (b) the system-2, excitation wavelength-778 nm. (c) the system-2 (annealed), excitation wavelength-776 nm. (d) Normalized luminescence comparison diagram at three different stages.

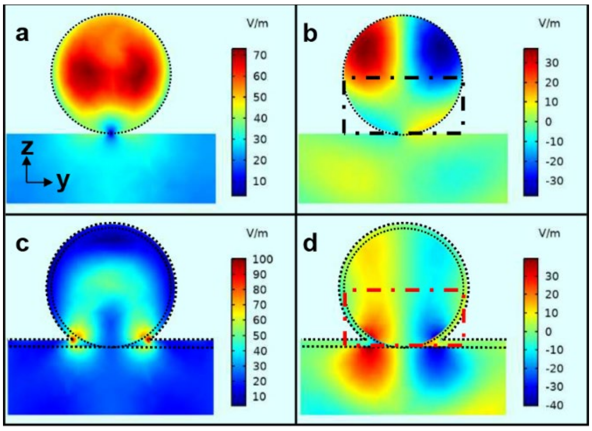

Figure S8: The simulated electric field distribution images. Distribution of electric field (a) and z component of electric field (b) of Si NS on Si substrate in YZ plane at the wavelength of 612 nm. Distribution of total electric field (c) and z component of electric field (d) of the system-2 in YZ plane at the wavelength of 612 nm.

The coupling property of the second sample corresponding to the system-2 is investigated also. The dependence of the up-converted luminescence on the excitation pulse power is plotted in a double-logarithmic coordinate to extract the slopes at three different stages. Before annealing, the extracted slope of the Si NS on Si substrate is 3.22 (Figure S9a), closing to 3, indicating that the emission of the 3PL is dominated. And the slope of the system-2 is 3.36 (Figure S9b) because there is a weak resonance coupling between the Si NS and WS_2 NMB. The slope of the system-2 (annealed) is 3.02 (Figure S9c). The SEM image (Figure S9c) shows the surface morphology of the second sample of system-2 (annealed) with d ~ 210 nm. It is noticed that the slope variation is inconsistent with that of the first sample of the system-2 (annealed) and the resonance valley occurs at the wavelength of 590 nm (Figure S9d) rather than that of 612 nm. The reasons for the discrepancy may be that the Si NS of system-2, by contrast, is not a perfect sphere and the WS_2 NMB is ablated to some extent due to the higher excitation power (Figure S9c, the red frame in the inset).

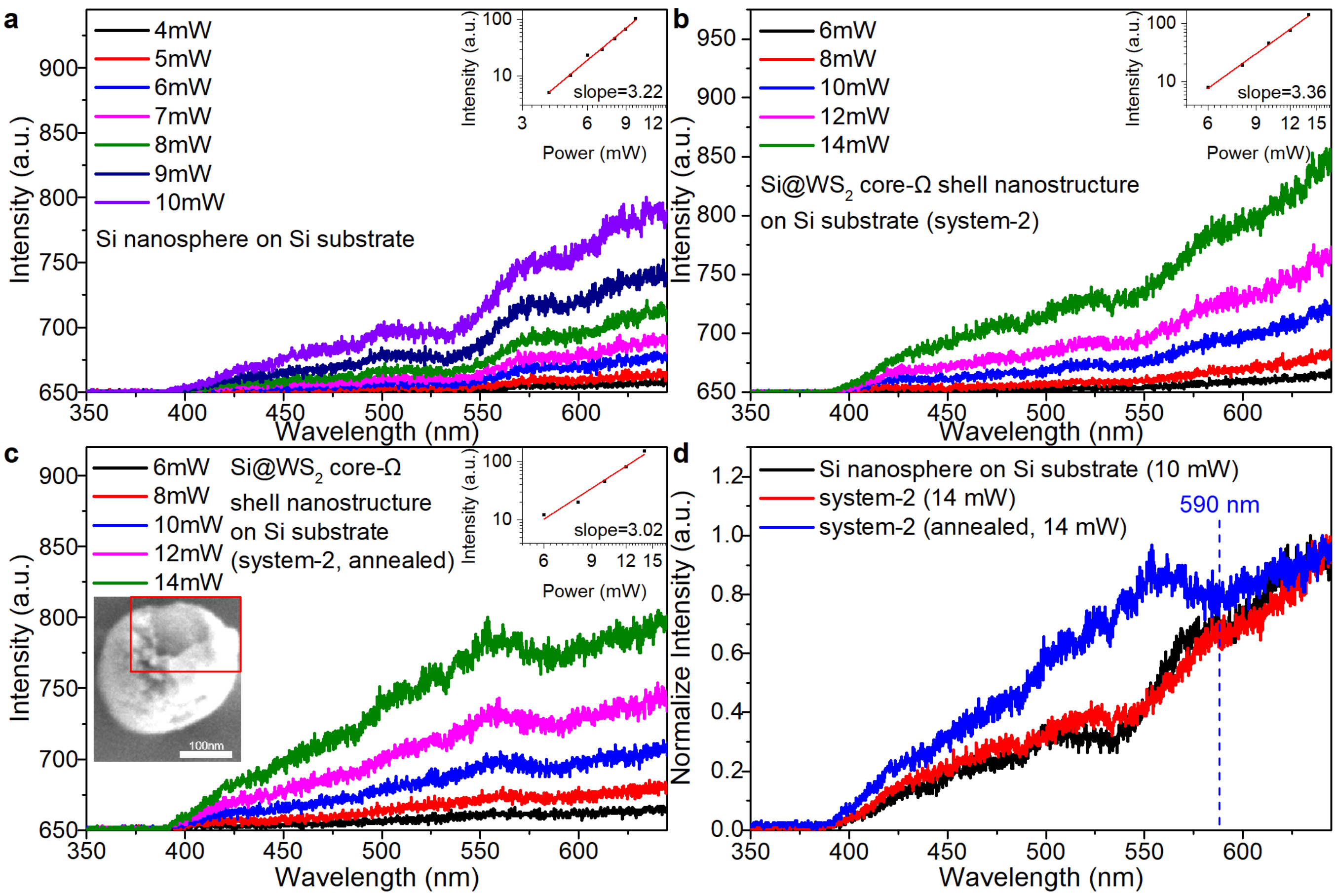

Figure S9. The dependence of the luminescence on excitation pulse power at three different stages. (a) the Si NS on Si substrate, excitation wavelength-777 nm. (b) the system-2, excitation wavelength-775 nm. (c) the system-2 (annealed), excitation wavelength-773 nm. (d) Normalized luminescence comparison at three different stages.
